# Case of Placenta Præmatura

**Published:** 1869-02-01

**Authors:** Ho. Berchelmann

**Affiliations:** Belleville, St. Clair County, Illinois


### *Case of Placenta Præmatura.*

BY HO. BERCHELMANN, M.D., BELLEVILLE, ST. CLAIR COUNTY, ILLS.

Was called on the night of the 14th to 15th of January, 1869, at 12.30 A. M., to attend Mrs. H. L., German, aged 36 years, a coal digger's wife, of light hair and complexion, and robust healthy constitution, in her eleventh confinement with her twelfth child. Found Mrs. St., a well instructed and intelligent midwife present. Learned from her that she had been sent for the same night after 10 o'clock, and found the woman suffering from extensive hæmorrhage, with the placenta presenting; she further said that the os was fully dilated, and I could begin to operate at once; of the child she could feel nothing.

The patient was restless and anxious, begged for help, had ineffective labor-pains and a weak, quick pulse, the blood streaming from under the bed. On examining with two fingers I found about two-thirds of the placenta filling the vagina, the rest retained and grasped as it were in the lower part of the uterus. It attracted my attention that the placenta was of loose, spongy and flabby structure, and not like a recently expelled healthy afterbirth, solidly bound together by the membranes; of the fœtus I was not able to detect anything. I immediately had the woman placed on a square bed, (the forceps being within reach if needed). I introduced my right hand, and by carrying it round between uterus and placenta disengaged the latter, and with a slight motion downward caused it to slip over the palm of the hand; with the last end of the placenta, and wrapped up in it, the right hand of the child descended into mine. When I separated the fœtal hand and placenta, the latter slipped and fell to the floor. In replacing the child's hand and arm I came in contact with the other (the left), evidently an entire square position of the child. In passing the child's arms and running my hand up along the cavity of the os sacrum, I reached the back of the fœtus, which convinced me at once that I had chosen the wrong hand, and that the child's feet must be found behind and above the arcus pubis. The uterus was in anteversio with the fundus, resting against the abdominal walls. I withdrew the right hand, with it elevated the uterus externally and straightened it, introduced the left hand behind the os pubis and immediately seized the child's right foot, effected version, and with careful rotary tractions delivered the whole fœtus. The cord was wound twice around the child's thorax, and I was much astonished to find that the placenta end of the cord was yet retained in utero. As I knew from the beginning the child to be dead I had until then paid no attention to the cord, and was under the impression that it had been torn when the placenta dropped out. Supposing now that a part of the placenta with the end of the cord was yet firmly attached to the uterus, I introduced the hand again and conveved it carefully around, sweeping the inner side of the uterus with the dorsal side of my hand; this motion, together with moderate traction at the cord, caused the latter to slip out with the membrames of the ovum at the end of it. The uterus now firmly contracted and the hæmorrhage ceased. I have seen the woman since several times, she is making a good recovery. At my last visit I learned from her, that on Sunday previous to her delivery she had a moderate flooding; the four days following she attended to her ordinary business without any outward symptoms, and on Thursday night at 8 o'clock she suddenly had severe hæmorrhage and slight labor-pains; at 10 o'clock she sent for the midwife. I think this is a decided case of placenta præmatura, and not of placenta prævia, properly speaking. My explanation is as follows:

On Sunday the detachment of the placenta commenced causing a moderate flow of blood; on Thursday, with the first labor-pains, the detachment was at once and entirely effected, followed by extensive hæmorrhage; the placenta was even separated on its fœtal side from the membranes, and by the contractions of the uterus expelled before the child. The membranes with the inserted end of the cord, by the pressure of the child's body, were glued to the inner wall of the uterus, and so retained. Had the child had a normal position and presented with the head, or even the nates, placenta and child would have been expelled together. It is perhaps proper to state here that fifteen months previous I attended the same woman when she had twins; the first child at that time was born alive at 9 A. M., head presenting, under the supervision of an ignorant self-made midwife, who did not send for me until six hours after the birth of the first child, and when the hand of the second was to be seen outside of the genital organ. with the cord prolapsed. I effected version and extraction. This second child of course was dead.

I wish to be excused if I have been rather lengthy in relating the foregoing case, bnt I consider it of sufficient interest and importance, especially for younger and less experienced members of the profession, who in case of emergency might be thrown entirely upon their own resources. Besides this I think it demonstrates the marked distinction between placenta præmatura and placenta prævia.

